# Tumour-associated circulating microparticles: A novel liquid biopsy tool for screening and therapy monitoring of colorectal carcinoma and other epithelial neoplasia

**DOI:** 10.18632/oncotarget.9018

**Published:** 2016-04-26

**Authors:** Arnulf Willms, Clara Müller, Henrike Julich, Niklas Klein, Robert Schwab, Christoph Güsgen, Ines Richardsen, Sebastian Schaaf, Marcin Krawczyk, Marek Krawczyk, Frank Lammert, Detlef Schuppan, Veronika Lukacs-Kornek, Miroslaw Kornek

**Affiliations:** ^1^ Department of General, Visceral and Thoracic Surgery, German Armed Forces Central Hospital, Koblenz, Germany; ^2^ Department of Medicine II, Saarland University Medical Center, Homburg/Saar, Germany; ^3^ Laboratory of Metabolic Liver Diseases, Department of General, Transplant and Liver Surgery, Medical University of Warsaw, Warsaw, Poland; ^4^ Department of General, Transplant and Liver Surgery, Medical University of Warsaw, Warsaw, Poland; ^5^ Institute of Translational Immunology and Research Center for Immune Therapy, Institute of Translational Immunology, University Medical Center, Johannes Gutenberg University, Mainz, Germany

**Keywords:** biomarker, CD147, CD326, CRC, diagnosis

## Abstract

Up to date, novel tools for low-cost, minimal invasive cancer surveillance, cancer screening and treatment monitoring are in urgent need. Physicians consider the so-called liquid biopsy as a possible future tool successfully achieving these ultimate goals. Here, we aimed to identify circulating tumour-associated MPs (taMPs) that could aid in diagnosing minimal-invasively the presence and follow up treatment in non-small cell lung carcinoma (NSCLC), colorectal carcinoma (CRC) and pancreas carcinoma (PaCa). Tumour-associated MPs (taMPs) were quantified after isolation by centrifugation followed by flow cytometry analysis from the serum of cancer patients with CRC (*n* = 52), NSCLC (*n* = 40) and PaCa (*n* = 11). Healthy subjects (*n* = 55) or patients with struma nodosa (thyroid nodules) (*n* = 43) served as negative controls. In all three types of tumour entities, the presence of tumour was associated with an increase of circulating EpCAM^+^ and EpCAM^+^CD147^+^ taMPs. The presence of CD147^+^EpCAM^+^ taMPs were specific to tumour-bearing patients thus allowing the specific distinction of malignancies from patients with thyroid nodules. Increased level of EpCAM single positive MPs were, in turn, also detected in patients with thyroid nodules. Importantly, EpCAM^+^CD147^+^ taMPs correlated with the measured tumour-volume in CRC patients. EpCAM^+^ taMPs decreased at 7 days after curative R0 tumour resection suggesting a close dependence with tumour presence. AUROC values (up to 0.85 and 0.90), sensitivity/specificity scores, and positive/negative predictive values indicated a high diagnostic accuracy of EpCAM^+^CD147^+^ taMPs. Taken together, EpCAM^+^CD147^+^ double positive taMPs could potentially serve as novel promising clinical parameter for cancer screening, diagnosis, surveillance and therapy monitoring.

## INTRODUCTION

Large cell membrane derived extracellular vesicles (EVs), known as microparticles (MPs), microvesicles (MVs) or ectosomes, have recently emerged as novel vehicles for a horizontal crosstalk between different cells and cell types, especially in the setting of inflammatory conditions [1–6]. In brief, MPs are extruded cell membrane coated vesicles with diameters between ~100–1000 nm that are formed and shedded during cellular activation or in early stages of apoptosis and that are released into the extracellular space. MPs can be isolated from human fluids such as whole blood, plasma, serum or e.g. synovial fluid [1–6]. They carry the surface signature of their cell of origin and the quantification of MP subsets using FACS sorting permits a non-invasive assessment of cell specific pathologies, especially in inflammation [7–10]. MPs have to be differentiated and separated from exosomes, which are derived from intracellular vesicles and do not carry cell surface markers of their origin, and from the larger fragments of apoptotic bodies [2, 3]. So far, only a few and technically limited studies have been performed on putative cancer-derived MPs or microvesicles identified by single surface marker [11–13]. Therefore, we explored the diagnostic potential of tumour-associated MPs (taMPs) and MP subtypes in thoroughly characterised patients with various underlying cancer entities such as colorectal carcinoma, non-small cell lung carcinoma and pancreas carcinoma.

## RESULTS

### Patients with CRC, other neoplasia or thyroid nodules (struma nodosa) show characteristic MP profiles

**Figure 1:**
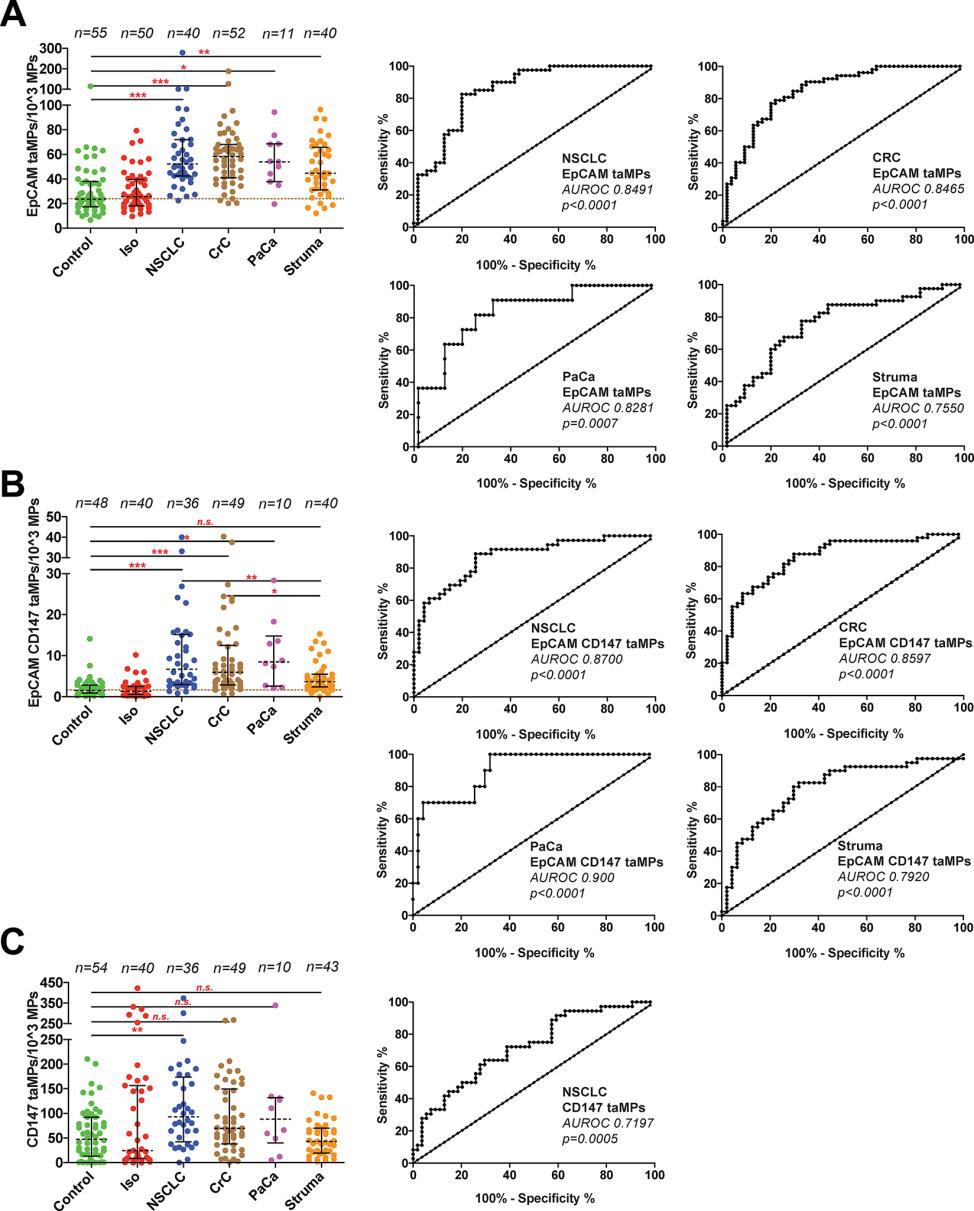
Detection of EpCAM^+^ and EpCAM^+^CD147^+^ and CD147^+^EpCAM^−^ tumour-associated microparticles (taMPs) in sera of indicated cancer patients (**A–C**) MPs were isolated by differential centrifugation and analysed by FACS as described in Supplementary. Materials and Methods. The indicated *p*-value for each MP population as predictor of controls vs. cancer and control vs. struma nodosa (thyroid nodules; short: struma) was calculated by using one-way ANOVA test including multiple comparisons using Dunn's post test. Shown are medians with 25 and 95 percentile. Additionally, accompanied areas under the receiver operating characteristics (AUROC) curves and values including accompanied *p*-values are shown as indicated; * = *p* < 0.05, ** = *p* < 0.005, *** = *p* < 0.0005, n.s. = not significant. Overall, an error level *p* < 0.05 was considered significant. Calculations were done with Prism 5 (GraphPad Software, Inc., USA).

Based on literature research various cancer markers were considered for the detection of taMPs expressing common cancer antigens as EpCAM and CD147. Corresponding cancer lines were screened for the chosen surface antigens (data not shown). Indeed, EpCAM and CD147 were identified on the surface of cancer cell lines of colorectal (CRC), lung (NSCLC) and pancreas (PaCa) (data not shown). MPs were isolated by differential centrifugation of sera of total 103 confirmed cancer patients. Median EpCAM^+^ taMPs values were significantly elevated (one-way ANOVA) in patients with CRC (*n* = 52), NSCLC (*n* = 40), PaCa (*n* = 11) by an average of 2.3 fold irrespective of the tumour entity and size (Figure 1A and Table 1). Surprisingly, EpCAM^+^ taMPs were also found elevated in thyroid nodules patients (short: struma, *n* = 40) as compared to healthy controls by 1.9 fold (*p* < 0.05). Nevertheless, the antigen combination of EpCAM and CD147, successfully detected *in vivo* derived CD147^+^EpCAM^+^ taMPs and their median values significantly increased in cancer patients by an average of 4.8 fold (Figure 1B and Table 1) across all investigated tumour entities. Additionally, the CD147^+^EpCAM^+^ taMPs were significantly reduced compared to the elevated cancer taMPs values (Figure 1B). However, *in vivo*, putative carcinoma derived single positive taMPs (CD147^+^EpCAM^−^) levels were not significantly distributed in our study (Figure 1C).

**Table 1:** Summary of predicted cut-off values, medians and other AUROC curve associated values of the indicated cancer entities

Cancer entity	Cut-Off taMPs/10^3^MPs	Median taMPs/10^3^MPs	Sensitivity [%]	Specificity [%]	AUROC	SD	*p*-Value
**NSCLC**	23.72*	52.28	97.50	50.91	0.8491	0.0388	< 0.0001
**CRC**	23.91*	58.21	94.23	52.73	0.8465	0.0373	< 0.0001
**PaCa**	23.72*	53.95	90.91	50.91	0.8281	0.0637	0.0007
**Control**		23.56					
**Cancer entity**	**Cut-Off taMPs/10^3^MPs**	**Median taMPs/10^3^MPs**	**Sensitivity [%]**	**Specificity [%]**	**AUROC**	**SD**	***p*-Value**
**NSCLC**	1.611^#^	6.72	91.67	55.32	0.8700	0.0392	< 0.0001
**CRC**	1.605^#^	5.93	95.92	55.32	0.8597	0.03771	< 0.0001
**PaCa**	1.611^#^	8.472	100.0	55.32	0.900	0.0479	0.0005
**Control**		1.48					

NOTE: Calculations were done with Prism 5 (GraphPad Software, Inc., USA) based on the measured individual taMP data of the indicated taMP populations. Overall, an error level *p* < 0.05 was considered significant.

* for EpCAM^+^ taMPs/1k MPs.

# for EpCAM^+^CD147^+^ taMPs/1k MPs.

### Diagnostic performance (AUROC)

For all investigated MP types, accompanied cut-off values were calculated based on their associated AUROC values (Table 1). The diagnostic performance of all three investigated taMPs populations (EpCAM^+^ taMPs, EpCAM^+^CD147^+^ taMPs and CD147^+^ taMPs) were assessed by their corresponding receiver operating characteristic (ROC) curves. The ROC curve is a plot of sensitivity versus 1- specificity for all possible cutoff values (Figure 1A–1C). Index of accuracy is the area under the ROC curve (AUROC). AUROC values reaching 1.0 indicating a high diagnostic accuracy. AUROC curves are given in Figure 1A–1C. EpCAM^+^CD147^+^ taMPs AUROC values were in general superior than the calculated AUROC value of EpCAM^+^ taMPs in this study (Table 1). CD147^+^ taMPs showed only a poor diagnostic relevance (AUROC data not shown).

### Positive (PPV) and negative predictive values (NPV) across cancer entities

Based on the overall cut-off value of 23.91 EpCAM^+^ taMPs/10^3^MPs, 99 out of 103 investigated cancer patients disregarding their cancer entity were correctly as tumour bearer identified and 90 out of 95 were identified as cancers by a cut-off value of 1.605 EpCAM^+^CD147^+^ taMPs/10^3^MPs. The overall positive (PPV) and negative predictivevalues (NPV) across the investigated cancer entities (NSCLC, CRC and PaCa) for EpCAM^+^ taMP were: 79.03% (PPV) and 85.29% (NPV), respectively, with an overall sensitivity of 95.15% and specificity of 52.73%. Individual sensitivities and specificities are depicted in Table 1. EpCAM^+^CD147^+^ taMPs were associated with a slightly higher overall positive predictive values for the investigated tumour entities: 80.36% (PPV) and 83.87% (NPV), respectively, with an overall sensitivity of 94.74% and specificity of 54.17%.

### Correlations between taMPs and CRC tumour load

**Figure 2:**
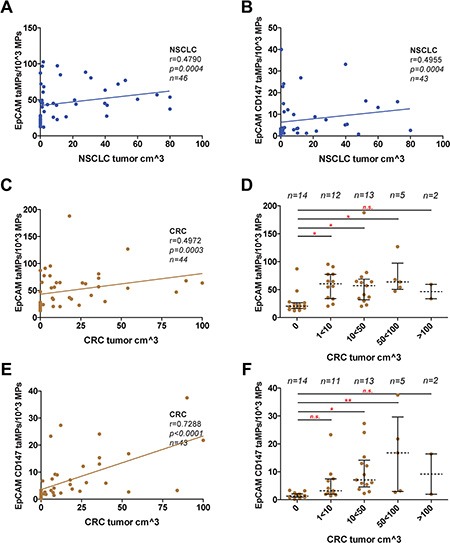
taMPs predict tumour volumes Measured EpCAM^+^ taMP and EpCAM^+^CD147^+^ taM*P* values were set in dependence (Spearman algorithm) to associate patient tumour volumes of indicated cancer entities; (**A–B**): NSCLC; (**C–F**): CRC. Correlations were restricted to 100 cm^3^ of tumour volume. (D/F) Detail analysis of indicated tumour ranges revealing the possible lower and upper detection limit. Shown are indicated median with 25 and 95 percentile including *p*-value as indicated; * = *p* < 0.05, ** = *p* < 0.005, *** = *p* < 0.0005, n.s. = not significant (one-way ANOVA test including multiple comparisons using Dunn's Multiple Comparison Test). Overall, an error level *p* < 0.05 was considered significant. Calculations were done with Prism 5 (GraphPad Software, Inc., USA).

**Figure 3:**
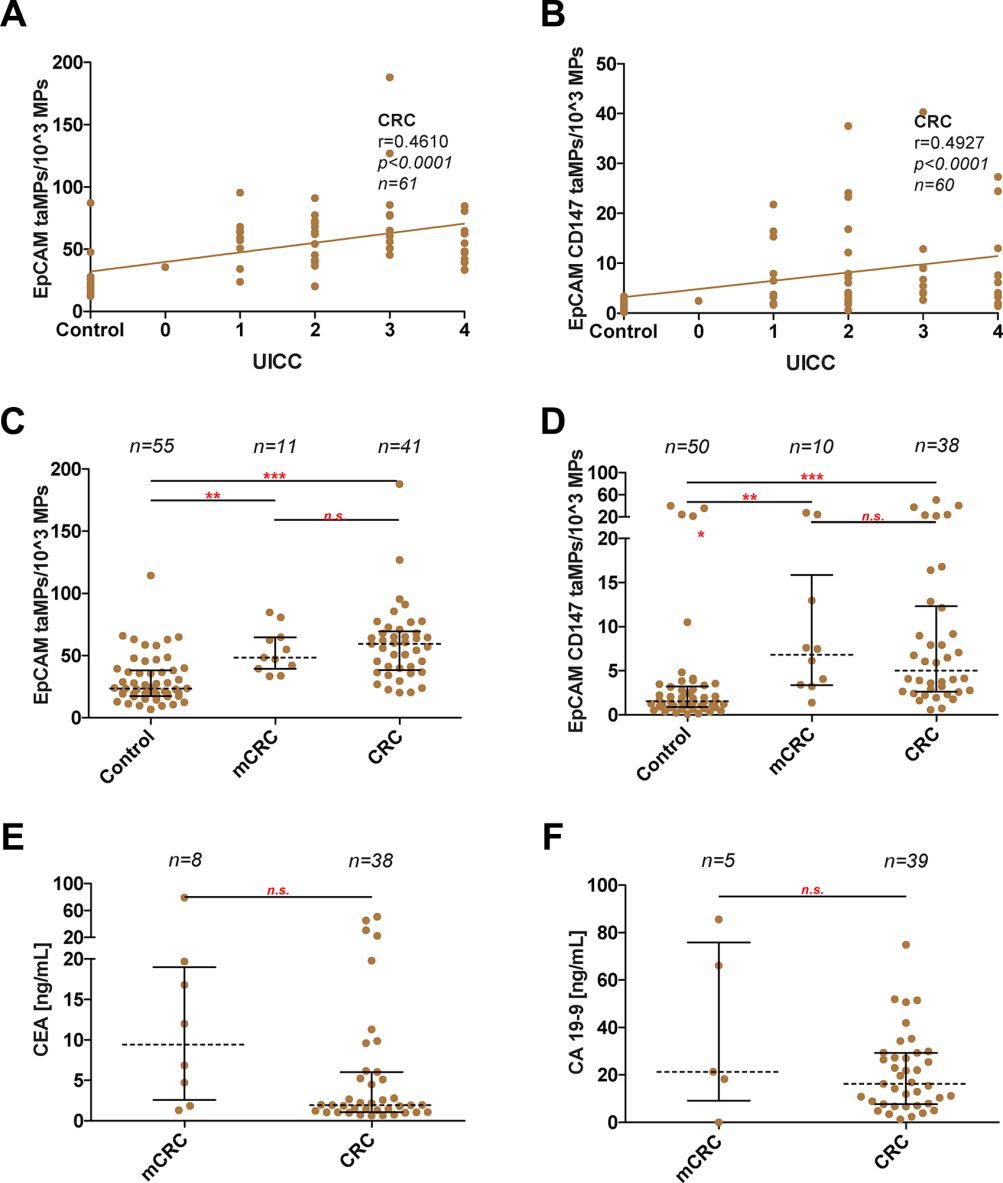
Discrimination between metastatic and non-metastatic phenotype in CRC (**A–B**) Measured EpCAM^+^ taMP and EpCAM^+^CD147^+^ taMP values were set in dependence (Spearman algorithm) to associate patient UICC values. (**C–D**) Direct comparison of indicated taMPs population in metastatic CRC (mCRC) vs. non-metastatic CRC and healthy controls. (E/F) Direct comparison of measured CEA and CA 19–9 values in ng/mL in metastatic CRC (mCRC) vs. non-metastatic CRC. Shown are indicated median with 25 and 95 percentile including *p*-value as indicated; * = *p* < 0.05, ** = *p* < 0.005, *** = *p* < 0.0005, n.s. = not significant (one-way ANOVA test including multiple comparisons using Dunn's Multiple Comparison Test (C/D) or unpaired Mann-Whitney test (E/F)).

While taMPs could aid in of the detection of the investigated tumour entities, the questions remained if taMPs, EpCAM^+^ taMPs and especially EpCAM^+^CD147^+^ taMPs could reflect tumour burden. However, in NSCLC we did not observe any correlation better than r = 0.5 between tumour volume and measured taMPs (Figure 2A–2B). In CRC, EpCAM^+^ taMPs were not indicating a sufficient correlation (r = 0.4972, Figure 2C–2D). But, as expected double positive taMPs, EpCAM^+^CD147^+^ taMPs, were correlating with CRC tumour volume significantly (r = 0.7288, *p* < 0.0001, *n* = 43) (Figure 2E). If CRC tumour volumes were broken up in meaningful tumour volume groups spanning from 0 (healthy controls), over 1–10 cm^3^, 10–50 cm^3^, 50–100 cm^3^ and above 100 cm^3^ CRC tumour volume, ANOVA analysis revealed that 10 cm^3^ of CRC volume might be the lower detection limit (Figure 2F). While a significant correlation between EpCAM^+^CD147^+^ taMPs and CRC tumour volume was observed, a good correlation exceeding r = 0.5 between EpCAM^+^CD147^+^ taMPs and EpCAM^+^ taMPs with the commonly used UICC scores for CRC were not reached (Figure 3A–3B). Leading to the further question whether was the shedding of taMPs from parental CRC tumour cells in fact dependent on the metastatic phenotype? Our data clearly indicated, that such dependence on the metastatic CRC phenotype wasn't given, there were no observed differences measured between EpCAM^+^ or EpCAM^+^CD147^+^ taMPs isolated from metastatic CRC patients samples or non metastatic CRC samples (Figure 3C–3D). Matched sera CEA and CA 19–9 sera parameters in those CRC samples from patients suffering metastatic or non-metastatic CRC showed no significant differences (*p* > 0.05) (Figure 3E–3F). Additionally no significant correlations between CRC tumour volumes and matched CEA (r = 0.2422, *p* = 0.06) or CA 19–9 (r = 0.059, *p* = 0.3601) sera values were observed.

### taMPs levels decrease after surgical CRC R0 resection

**Figure 4:**
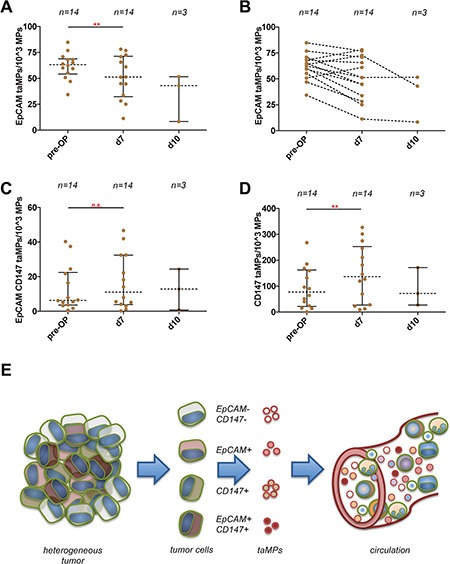
Detection of EpCAM^+^ and EpCAM^+^CD147^+^ and CD147^+^EpCAM^−^ tumour associated microparticles (taMPs) in CRC sera patient samples (**A**) EpCAM^+^ taMPs levels 7 days post CRC tumour resection and at day 10 post-OP. (**B**) Paired display of accompanied post-OP and post-OP values of indicated taMP populations. (**C**) EpCAM^+^CD147^+^ taMPs levels 7 days post CRC tumour resection and at day 10 post-OP. (**D**) CD147^+^EpCAM^−^ taMPs levels 7 days post CRC tumour resection and at day 10 post-OP. Shown are indicated median with 25 and 95 percentile including *p*-value as indicated; * = *p* < 0.05, ** = *p* < 0.005, *** = *p* < 0.0005, n.s. = not significant (paired *t*-test with Wilcoxon matched pairs signed rank-test). Overall, an error level *p* < 0.05 was considered significant. (**E**) Sketch illustrating the heterogeneous tumour composition and tumour antigens used in the current study. Tumour cells shed multiple MP subpopulations carrying a distinguishing set of surface markers. These shedded taMPs can be detected in the sera of cancer patients.

Furthermore, EpCAM^+^ taMPs values were evaluated in serum of CRC patients at pre- and post-surgery stages (pre-OP, usually the day before the planed R0 CRC resection, *n* = 14; d7, post-operative day 7, *n* = 14; d10, post-operative day 10, *n* = 3). In total, 11 out of 14 resected CRC patients with longitudinal blood collections showed a significant decrease from pre-OP 61 EpCAM^+^ taMPs per 10^3^ AnnexinV^+^ MPs to 51 EpCAM^+^ taMPs per 10^3^ AnnexinV^+^ MPs (Figure 4A–4B). On the contrary, EpCAM^+^CD147^+^ taMP and CD147^+^EpCAM^−^ MPs were at day 7 post-OP in median not differing compared to respective pre-O*P* values (*p* > 0.05) (Figure 4C–4D).

## DISCUSSION

A recent study demonstrated that analysis of blood exosomes, another kind of small sized extracellular vesicles of 30–100 nm size that do not entirely share the same surface markers with MPs, might help to diagnose non invasively patients with pancreatic ductal adenocarcinoma (PADC) [14]. Based on the well founded hypothesis that cell specific MPs (200–1000 nm in size) are released into the circulation as a result of activation and/or apoptosis of their parent cell type [7], we searched for EpCAM^+^, CD147^+^ or EpCAM^+^CD147^+^ double positive taMPs that could indicate cancer presence.

Depending on the overall cut-off value 99 out of 103 investigated cancer patients disregarding their cancer entity were correctly as tumour bearer identified and 90 out of 95 were identified as cancers by EpCAM^+^CD147^+^ taMPs. Additionally, the calculated overall positive (PPV) and negative predictive values (NPV) across the investigated cancer entities (NSCLC, CRC and PaCa) for EpCAM^+^CD147^+^ taMPs distinguishing CRC and other neoplasia from healthy controls and from thyroid nodules (*struma nodosa*). These results suggest that tumours can be reliably detected with taMP profiling including EpCAM^+^ taMPs and EpCAM^+^CD147^+^ taMPs independently of the underlying cancer entity. However, certain disease/health circumstances associated with epithelial damage such as struma nodosa (thyroid nodules) does not permit to draw conclusions only on EpCAM taMPs. Thus, such disorders causing epithelial damage should be regarded as exclusion criteria in MP-based studies.

Next, we investigated whether both taMP populations, would reflect the tumour volume. For NSCLC only poor correlations (r < 0.5) were observed between the investigated taMPs populations and the tumour volume (Figure 2A–2B). EpCAM^+^ taMPs could not indicate tumour volume in CRC (Figure 2C). However, EpCAM^+^CD147^+^taMPs were greatly mirroring tumour volume with a significant dependence (r = 0.73; Figure 2E). Furthermore the detailed analysis revealed that in CRC the tumour volume of less than 10 cm^3^, (~2 cm in diameter) might be the lowest taMP detection limit (Figure 2F).

EpCAM is currently used as pan-cancer marker and their expression is also associated with stem cells [15, 16]. Additionally, EpCAM is a prominent cell surface antigen for detection of circulating tumour cells (CTCs) and part of the commercially available CellSearch™ system (Veridex LLC, Raritan, NJ, USA). Of note, the US Food and Drug Administration (FDA) approve this test only for the detection of certain metastatic cancers [17–19] whereas in our novel approach we cannot observe such limitation for either of the investigated taMPs subpopulations (Figure 3C–3D).

Next we explored whether total tumour R0 resection could alter taMPs. Based on the previously published results [14], we expected that taMPs should decrease after tumour removal. Indeed, we documented a significant decrease of the median values at day 7 post-OP from pre-OP 61 EpCAM^+^ taMPs per 10^3^ AnnexinV^+^ MPs to 51 EpCAM^+^taMPs per 10^3^ AnnexinV^+^ MPs (Figure 4A–4B). While EpCAM^+^ taMPs did not decrease totally towards the healthy control levels. We assumed that the clearance of EpCAM^+^ taMPs might need longer time period as in the case of the clearance of Glypican-1 positive exosomes [14]. On the contrary, EpCAM^+^CD147^+^ taMP and CD147^+^EpCAM^−^ MPs were at day 7 post-OP in median not differing compared to respective pre-O*P* values (Figure 4C–4D). We speculate that CD147^+^EpCAM^−^ MPs were shed from fibroblast, T-cells, stroma cells, epithelial cells during tumour resection indicating tissue remodelling, migration and cancer cell invasion in which EMMPRIN/CD147 might play role as suggested by others [7, 20, 21].

Taken together, *in vivo* cancer cells shed distinct taMP populations with a unique pan-cancer MPs-based signature. Even if each tumour is composed of a mixture of heterogeneous tumour cells, the released EpCAM^+^ and EpCAM^+^CD147^+^ taMPs can be reliable detected in the circulation in both primary and metastatic tumour-bearing patients (Figure 4E). EpCAM^+^ and EpCAM^+^CD147^+^ taMPs might serve as an early indicator of cancer growth and monitor successful anti-tumour therapy and might be used as important liquid biopsy tool to differentiate between therapy responders and therapy non-responders. Importantly, our data clearly indicate that it is far more beneficial to explore specific individual taMP subpopulations using multiple surface marker combination in order to distinguish cancer from non-cancer patients. Our results demonstrate that the analysis of taMP can help to identify patients with cancer from healthy individuals but also to pinpoint the presence of specific MP subtype in epithelial damage and subtype exclusively associated with tumour.

## MATERIALS AND METHODS

### Human study cohort

The Ethics Commission of the State Chamber of Medicine in Rhineland-Palatinate approved the current study (approval number: 837.151.13 (8836-F)) and all patients gave their informed consent prior to participation. Patients with a major second or third known comorbidity that could affect immune cell activation such as acute inflammation, chronic inflammation, autoimmune diseases or viral infections, were excluded. Additionally, patients who underwent chemotherapy or were receiving chemotherapy or were subjected to any other anti-tumoural therapy during the time blood samples were taken were excluded, too. Characteristics of patients are summarized in Supplementary Table 1.

### Isolation of cell derived microparticles from human serum

Blood was collected in standard S-Monovette^®^ 7.5 ml, Serum Gel with Clotting Activator (Sarstedt AG & Co., Nümbrecht, Germany) and left for 30 minutes at 37°C to allow for clot formation followed by centrifugation at 4000 rpm for 20 min at 4°C. Clots were carefully separated and supernatants stored at −80°C for further MP isolation. MPs from serum samples were isolated by differential centrifugation between 2,000 and 20,000 g as described by us and others [4, 5, 7]. MPs sedimenting at 20,000 g were characterized by FACS using staining for AnnexinV, CD147, EpCAM (eBioscience™, San Diego, CA; BioLegend; Miltenyi Biotec, Bergisch Gladbach, Germany, respectively). All antibodies were titrated against the matching isotype control on patient's samples prior to use. MP preparations were characterized on a MACSquant 10 Analyser (Miltenyi Biotec, Bergisch Gladbach, Germany) and cytometric data was analysed with FlowJo X software for MAC OSX (Tree Star, Inc., Ashland, Oregon). To avoid non-specific antibody binding, Fc receptors on MPs and target cells were blocked with FcR Blocking Reagent (eBioscience™, San Diego, CA, USA). Additionally, MPs were dialyzed overnight against AnnexinV binding buffer containing 0.05% BSA (Miltenyi Biotec, Bergisch Gladbach, Germany). Used antibodies and BSA blocking solution were centrifuged prior to FACS to avoid artefacts due to aggregation. No differences regarding the total amount of MPs isolated were observed between healthy and cancer samples (Supplementary Figure 2).

### Statistical analysis

All data are medians with their percentile. Differences between independent experimental groups (NSCLC, CRC and controls) were characterized using the one-way ANOVA test. As a post-hoc test, a Dunn's test was applied for multiple comparisons of subgroups when the one-way ANOVA test was positive and succeeded Bartlett's test for equal variance. A two-tailed Wilcoxon matched pairs signed rank-test was applied to assay differences between patients who underwent a total R0 tumour resection between pre-operative and postoperative sera samples. To assess the predictive ability of the two taMP populations (EpCAM^+^ and EpCAM^+^CD147^+^ taMPs) and for discriminating between individuals with cancer, struma nodosa (thyroid nodules) and controls, we calculated sensitivity, specificity, PPV and NPV and AUROC values. These calculations were done with Prism 5 (GraphPad Software, Inc., USA). Overall, *p* < 0.05 was considered significant. The overall power (1-β err prob) of the study was calculated post-hoc with GPower (Version 3.1.9.2) assuming a minimum effect size of f = 0.5 with an α err prob = 0,05 for 5 groups, indicating that the minimum total sample size of 80 is needed to reach a power of 0.95 (1-β err prob).

## SUPPLEMENTARY MATERIALS FIGURES AND TABLE



## Abbreviations

AUROC: Area under the Receiver Operating Characteristic
CTC: circulating tumour cell
CRC: colorectal carcinoma
EpCAM: epithelial cell adhesion molecule
MP: microparticle
NPV: negative predictive value
PPV: positive predictive value.
