# Supplementary material for: Tumour-associated circulating microparticles: A novel liquid biopsy tool for screening and therapy monitoring of colorectal carcinoma and other epithelial neoplasia
Source: Oncotarget. 2016 Apr 26;7(21):30867–75. doi: 10.18632/oncotarget.9018 (PMC5058724; doi:10.18632/oncotarget.9018)
Supplement: Supplementary file 1 [file oncotarget-07-30867-s001.pdf]

# Tumour-associated circulating microparticles: a novel liquid biopsy tool for screening and therapy monitoring of colorectal carcinoma and other epithelial neoplasia

## Supplementary Materials

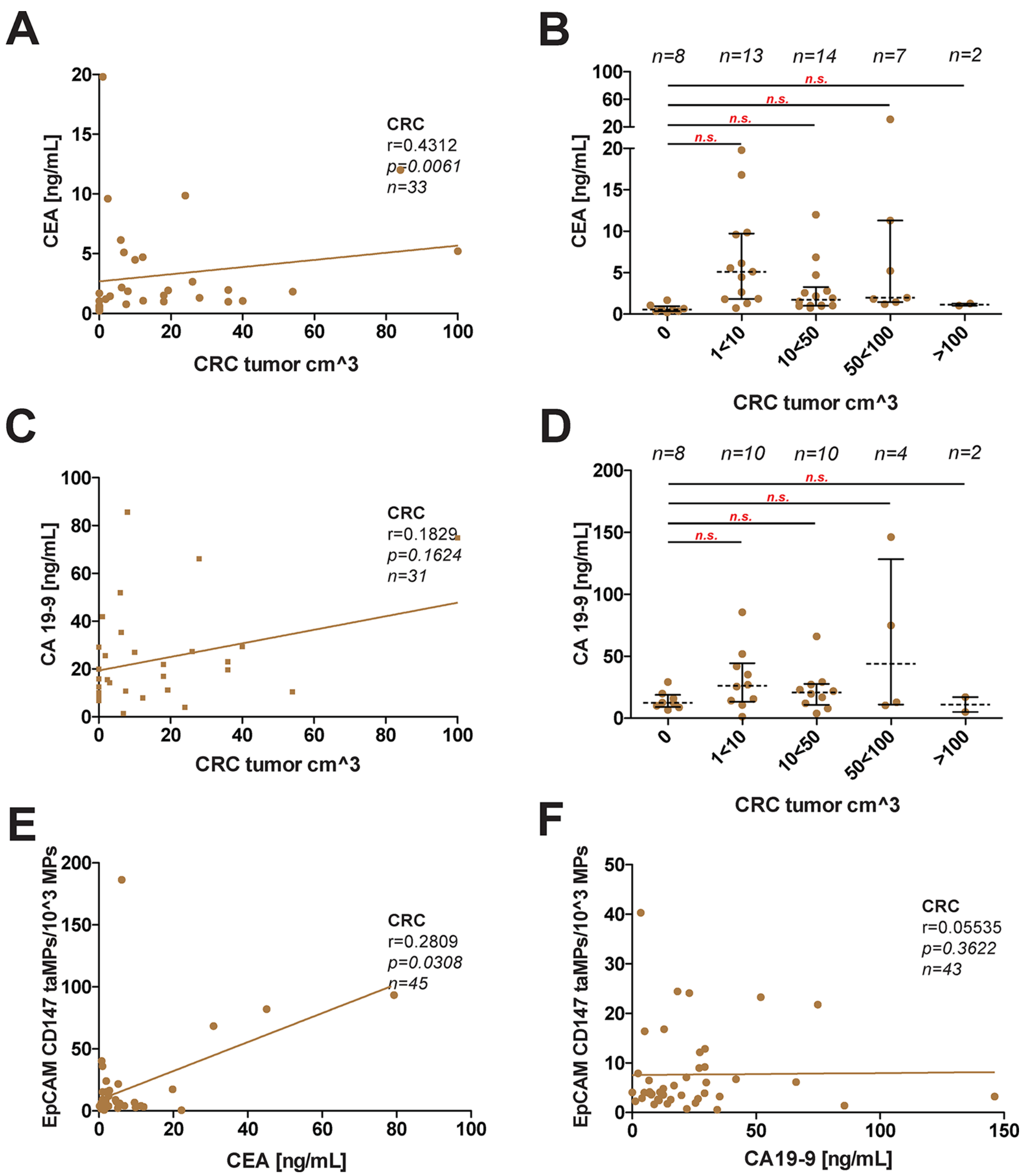


**Supplementary Figure S1: CEA and CA19-9 as predictor of actual tumour volume in CRC.** (**A–D**) Measured CEA and CA 19–9 values were set in correlation (Spearman algorithm) to associate patient's tumour volumes. Correlations were restricted to 100 $cm^3$ of tumour volume. (B, D) Detailed analysis of indicated tumour ranges revealing the possible lower and upper detection limit. (**E**, **F**) Dependence between indicated taMPs and CEA or CA19-9 patient sera values. Shown are indicated median with 25 and 95 percentile including *p*-value as indicated; * = $p < 0.05$, ** = $p < 0.005$, *** = $p < 0.0005$, n.s. = not significant (one-way ANOVA test including multiple comparisons using Dunn's Multiple Comparison Test).

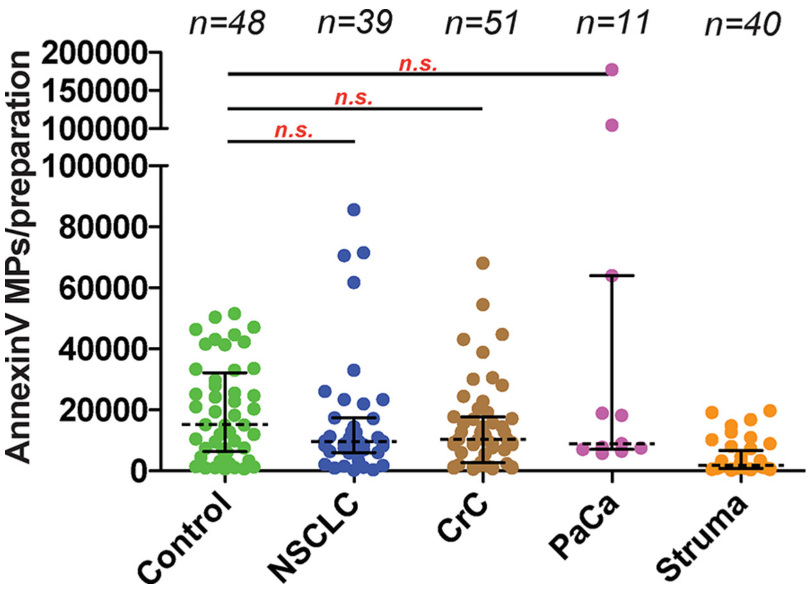


**Supplementary Figure S2: Total AnnexinV values per MP isolation from human sera samples.** Overview of the measured total AnnexinV values from each human sera preparation with the indicated cancer entity or control. Shown are indicated median with 25 and 95 percentile including *p*-value as indicated; * = $p < 0.05$, ** = $p < 0.005$, *** = $p < 0.0005$, n.s. = not significant (one-way ANOVA test including multiple comparisons using Dunn's Multiple Comparison Test). NOTE: struma sera volumes were less than 1mL, therefore total MPs/preparation are lower in these samples.

## Supplementary Table S1: Summary of demographic, biochemical and histological parameters of patients with indicated tumour entity

| | CRC | NSCLC | PaCa | Struma | Controls |
|---|---|---|---|---|---|
| **Patients [#]** | 52 | 40 | 11 | 43 | 55 |
| **Female [#]** | 17 | 9 | 7 | 31 | 19 |
| **Male [#]** | 35 | 31 | 4 | 12 | 36 |
| **Age [years]** | 66.60 | 64.01 | 67.31 | 55.48 | 27.52 |
| ***Tumour volume [cm$^3$]** | 36.41 | 31.50 | 34.10 | n.a. | 0.00 |
| ***CEA [ng/mL]** | 7.587 | 6.730 | 2.607 | n.a. | 0.679 |
| ***CA19-9 [ng/mL]** | 22.067 | n.a. | n.a. | n.a. | 14.46 |
| ***UICC I** | 10 | 15 | 2 | 0 | 0 |
| ***UICC II** | 14 | 9 | 3 | 0 | 0 |
| ***UICC III** | 12 | 5 | 2 | 0 | 0 |
| ***UICC IV** | 11 | 7 | 7 | 0 | 0 |
| ***Tumour resected** | | | | | |
| **yes** | 42 | 28 | 8 | 0 | 0 |
| **no** | 10 | 9 | 2 | 43 | 55 |
| ***R0 resection** | 33 | 24 | 5 | 0 | 0 |
| ***Neoadjuvant radio-/ Chemotherapy** | 16 | 5 | 0 | 0 | 0 |

NOTE: #, absolute number of patients in each cohort; age, CEA- and CA19-9 values are given in ng/mL as means; calculated mean tumour volume values are shown in cm$^3$ according to MRI criteria.; n.a: not available; *: if available.
